# Immunity to rubella: an Italian retrospective cohort study

**DOI:** 10.1186/s12889-019-7829-3

**Published:** 2019-11-08

**Authors:** Francesco Paolo Bianchi, Sara De Nitto, Pasquale Stefanizzi, Angela Maria Vittoria Larocca, Cinzia Annatea Germinario, Silvio Tafuri

**Affiliations:** 1 Department of Biomedical Science and Human Oncology, “Aldo Moro” University of Bari, Piazza Giulio Cesare 11, 70124 Bari, Italy; 2 Hygiene Department, Bari Policlinico General Hospital, Bari, Italy

**Keywords:** Healthcare workers, Booster dose, Duration of immunization, MMR vaccine

## Abstract

**Background:**

International guidelines recommend that healthcare workers (HCWs) have presumptive evidence of immunity to rubella and that susceptible HCWs and doubt cases receive two doses of the MMR vaccine. However, a small percentage of the fully immunized will remain unprotected against wild viruses. Moreover, protective levels of antibodies induced by the vaccine have been shown to decline over time, but a formal recommendation regarding the testing of immunized HCWs for the persistence of IgG against rubella is lacking.

**Methods:**

The aim of this study was to evaluate the long-term immunogenicity conferred by rubella vaccination and the effectiveness of a strategy for the management of immunized individuals in whom IgG against rubella could not be demonstrated (non-responders). The study enrolled students and medical residents who attended the Hygiene Department of Bari Policlinico University Hospital for biological risk assessment (April 2014 to June 2018).

**Results:**

Two thousand students and residents with documented immunization (≥2 doses of rubella or MMR vaccine) were tested. In 181 (9%), IgG against rubella was not detectable. The seronegative rate was higher among participants vaccinated at age < 2 years (89.6%) and lower among those immunized at age ≥ 2 years (93.6%; *p* < 0.0001). The administration of a single MMR booster dose resulted in a seroconversion rate of 98% in the seronegative group. The seroconversion rate after a second booster dose was 100%. No serious adverse events in the re-immunized were recorded.

**Conclusions:**

An important proportion of individuals immunized for rubella or MMR do not have a protective titer for the disease(s). Our management strategy (booster followed by re-test and, for those who are still negative, a second booster and re-test) is consistent with the goal of achieving immunological memory.

## Background

Rubella is a viral-vaccine-preventable disease but adverse effects may occur in non-vaccinated infants and adults. In non-immunized pregnant women, infection may also pose a high risk of fetal complications. The WHO reported that in 2017 there were 16,391 cases of rubella worldwide [1]. International public health institutions have set a goal of eliminating measles and rubella in at least five WHO regions by the end of 2020 [2], mainly by achieving a high vaccine coverage [3]. Rubella immunization campaigns are currently based on the use of different types of vaccines: the monovalent anti-rubella vaccine (not available in Italy), the MMR (measles, mumps, rubella) vaccine and the MRPV (measles, mumps, rubella, varicella) vaccine, all of which contain live-attenuated virus. The MMR and MRPV vaccines provoke an adequate immune response simultaneously for the three/four viruses and thus facilitate the implementation of current immunization strategies [3].

According to pre-licensure data, one dose of MMR vaccine is 97% effective in the prevention of rubella and two doses are ~ 99% effective [4]. The seroconversion rate for rubella after a single dose is 95% [5]. Based on the evidence obtained since the introduction of global mass vaccination, the MMR vaccine is safe [6], cost-saving [7], and effective [4].

Since 2003, Italian national vaccination policy has included universal mass vaccination for measles, mumps and rubella using two doses of the MMR vaccine, in accordance with the recommendations of the US Center for Disease Control and Prevention (CDC) [4]. In 2017, the Italian government made rubella vaccination compulsory for infants and teenagers [8]. Although this vaccination strategy was very effective, rubella has yet to be eliminated, due to a vaccine coverage that is suboptimal [9] and far below the level conferring critical coverage (≥95%) as defined in national and international plans [10]. Indeed, from 2015 to 2018 there were 88 recorded cases of congenital rubella in Italy, including 22 infants who developed chronic heart, eye and/or acoustic disease, and 173 cases of rubella during pregnancy (median age at pregnancy: 27-years). Three women who developed rubella during pregnancy were subsequently vaccinated against the virus [11].

The CDC recommends that healthcare workers (HCWs) have presumptive evidence of immunity to rubella [12], based on documented vaccination or a history of natural disease. This recommendation is crucial for certain subgroups of HCWs, such as those who work in Obstetric Departments. In the “post-vaccination era,” many seroprevalence studies have described a notable percentage of HCWs susceptible to rubella (12.8%), related to a missed vaccination or waning IgG levels after immunization [13]. Non-seroprotected HCWs are an important public health issue, as they represent a risk both for themselves and for patients [14]. Although the lack of seroprotection mostly involves unvaccinated HCWs, infectious diseases may also develop in those who are vaccinated. However, this issue remains poorly investigated.

Rubella immunity is commonly considered to be life-long, but several studies have shown a decline in the levels of rubella antibodies over time and that immunity induced by successful primary immunization lasts only for 15–20 years [12]. Furthermore, the effectiveness and long-term immunogenicity of the vaccine may be influenced by the immunization strategy, as recently shown for pertussis [15]. In particular, the absence of a natural booster may lead to a decline of the IgG level in a fully vaccinated person.

The aim of this study was to evaluate the long-term immunogenicity conferred by rubella vaccination and the impact of an immunization strategy in the management of non-responders, i.e., immunized individuals who nonetheless lack IgG against rubella.

## Methods

Our study was carried out in Apulia, Southern Italy (~ 4,000,000 inhabitants), where currently there are no formal regulations regarding vaccination or determination of the immune status of HCWs at high risk of rubella circulation [16, 17]. As mandated by the Italian Ministry of Health, students at medical schools as well as medical students and residents at the university hospital are subject to the same procedures specified by Italian law regarding the occupational health and safety of HCWs [18]. Therefore, in April 2014, the Hygiene Department of the Bari Policlinico University Hospital implemented a biological risk prevention program for students and residents of the medical school of the University of Bari.

Ours was a retrospective cohort study of students and residents who attended the Hygiene Department from April 2014 to June 2018. During this period, the data of each screened individual obtained during the initial interview were entered into a computerized registry. For the purposes of our investigation, only students and residents who at the time of enrollment had received 2 doses of rubella/MMR vaccine (basal vaccine cycle) were included in the study. Students and residents without an available vaccination history, who were never vaccinated, who were vaccinated with a single dose of rubella/MMR vaccine at baseline, or who had a history of rubella infection were excluded from the analysis.

Since non-seroprotection in the previously immunized has been poorly investigated, we could not formulate a hypothesis regarding the prevalence of vaccinated seronegative individuals and were thus also unable to calculate the adequate sample size. However, our study consisted of a very large number of participants to support the validity of its findings.

The vaccination status of the study participants was assessed using the Regional Immunization Database (GIAVA) [18], a computerized vaccination registry that contains information on the vaccination history and immunization schedule of every Apulian inhabitant.

For each enrollee, a 5-mL serum sample was collected to assess the rubella immunity/susceptibility status, determined by a chemiluminescence assay. Individuals whose anti-rubella IgG titer was > 10 IU/mL were considered seroprotected, those with a titer < 7 IU/mL as non-seroprotected, and with a titer of 7–10 IU/mL as having an equivocal seroprotection status.

**Fig. 1:**
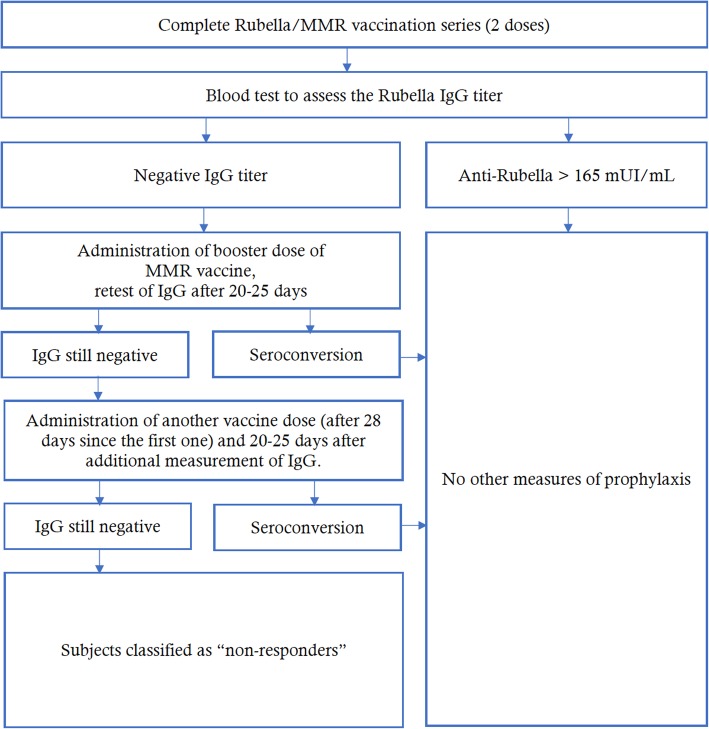
Assessment of the biological risk for rubella in study participants who had undergone the basal vaccination series (2 doses of MMR vaccine)

Individuals in the seronegative group received a first booster dose of MMR vaccine (M-M-RVAXPRO, administered subcutaneously in the deltoid). Those with equivocal tests were re-tested; if the results were still equivocal the individual was classified as negative and then they received a second booster dose. Twenty to 25 days after the vaccination, a new blood test was performed to measure IgG titers; if the value exceeded the cut-off, the person was classified as seroconverted; if the titer was still negative, another vaccine dose (28 days after the first booster) was administered and 20–25 days thereafter the IgG level was measured again. Non-responders were those who remained seronegative after two booster doses (Fig. 1). This management strategy complied with the protocols applied at a US Medical School [19]. Participants who received the booster doses underwent a 1-month follow-up to assess any adverse effects.

For every participant, the following information was obtained: i.d., sex, age at enrollment, dates of the routine MMR vaccine, rubella IgG titer, date of the first booster, IgG titer after the first booster, date of the second booster, IgG titer after the second booster. The information was entered into an Excel spreadsheet and the data were analyzed using STATA MP15 software.

Continuous variables are reported as the mean ± standard deviation and range, categorical variables as proportions, with the 95% confidence interval (95%CI) when appropriate. The Wilcoxon’s rank sum test was used to compare continuous variables between enrollees who received the first dose of a routine MMR vaccine at age < 2 years and those who received the first dose at ≥2 years. The chi-squared test and Fishers exact test were used to compare proportions. Although according to the current vaccination schedule the first dose of the MMR vaccine should be administered within the first 13–15 months of life, we set a cut-off of 23 months (typically the case in the general population, given the common delay in the vaccination appointment) and asked whether the response of this group differed from that of the group vaccinated with the first dose at an older age.

A univariate logistic regression was used to investigate the determinants of seroconversion after the basal vaccine cycle, considering seroconversion as outcome and sex, age at enrollment, age at the time of the first vaccination of the basal cycle, age at the time of the second vaccination of the basal cycle, time from the first to the second vaccination in the basal cycle, time from the first vaccination to the measurement of the antibody titer and the time from the second vaccination to the measurement of the antibody titer as determinants. The odds ratio (OR) was calculated together with the 95%CI and was followed by a z-score test.

For each of the previous outcomes, a multivariate logistic regression model was constructed in which the age at the first dose of routine vaccination (< 2/≥2 years) served as the determinant, adjusted for the variables identified in the univariate logistic regression. The adjusted odds ratio (aORs) was calculated together with the 95%CI and was followed by a z score test.

The determinants of seroconversion after a booster dose were identified in a univariate logistic regression, considering seroconversion after the booster doses as the outcome and sex, age at enrollment, age at the time of the first vaccination of the basal cycle, age at the time of the second vaccination of the basal cycle, time from the first to the second vaccine in the basal cycle, time from the first vaccine of the basal cycle to the booster dose, time from the second vaccine of the basal cycle to the booster dose, and the time from the booster dose to the antibody re-titer evaluation. The OR and 95%CI were calculated, followed by a z-score test.

For each of the previous outcomes a multivariate logistic regression model was constructed in which the age at first vaccine of the routine vaccination (< 2/≥2 years) served as the determinant, adjusted for the variables identified in the univariate logistic regression. The aOR was calculated together with the 95%CI, followed by a z score test. Pearson’s chi-squared test was used to evaluate the goodness of fit of the multivariate logistic regression models.

Protective antibody survival (PAS) was evaluated as well, defined as the time elapsed from the measurement of the antibody titer to the second dose of the routine MMR vaccine.

Kaplan-Meier curves were used to evaluate PAS, and the log-rank and Breslow tests to evaluate the differences between groups. The median PAS time and the incidence rate per person-year for the loss of seroprotection were estimated. The incidence rate ratio (IRR) was calculated, considering individuals vaccinated at < 2 years as not exposed and those vaccinated at ≥2 years as exposed.”

A univariate Cox semiparametric regression was used to evaluate the determinants of PAS, with sex, age at enrollment, age at the second vaccine of the routine vaccination and age at the time of the first vaccination of the basal cycle (< 2/≥2 years) as the risk predictors. Based on the outcome, a multivariate Cox semiparametric regression model was constructed in which the determinants from the univariate regression served as the risk predictors. Schoenfeld and scaled Schoenfeld residuals tests were used to analyze the proportionality assumption of the multivariate Cox semiparametric regression model.

For all analyses, a *p*-value < 0.05 was considered statistically significant.

The study was conducted in accordance with the principles of the World Medical Association Declaration of Helsinki and did not involve any experiments on humans or human samples nor research on identifiable human material or data.

## Results

From April 2014 to June 2018, 4563 students and residents were tested. A vaccination certificate was available for 4225/4563 (92.6%) participants, and 2000/4225 (47.3%) had a complete rubella/MMR vaccination schedule. In the latter group, 1387 (69.4%) were female.

A first routine vaccine was administered at age < 2 years to 1330 of the 2000 (66.5%) participants and at age ≥ 2 years to 670/2000 (33.5%). The proportion of females in the group immunized at < 2 years (*n* = 930/1330; 69.9%) vs. at ≥2 years (*n* = 458/670; 68.2%) was not significantly different (X^2^ = 0.6; *p* = 0.432). The average age at study enrollment was 21.1 ± 2.4 years (range = 18.0–38.0), with a slight difference between the group vaccinated at < 2 years (20.8 ± 2.1; range = 18.0–35.0) and at ≥2 years (21.8 ± 2.8; range = 18.0–38.0; z = 8.4; *p* < 0.0001).

All study enrollees with a complete baseline vaccination cycle were tested for anti-rubella IgG. None reported a history of rubella.

A protective anti-rubella IgG titer was determined in 1819/2000 (91.0%; 95%CI = 89.6–92.2%) but the proportion was lower among individuals vaccinated at < 2 years (*n* = 1192/1330; 89.6%; 95%CI = 87.9–91.2%) than at ≥2 years (*n* = 627/670; 93.6%; 95%CI = 91.5–95.3%; *X*^2^ = 8.5; *p* = 0.004). The overall geometric mean of the anti-rubella IgG titer was 32.4 (95%CI = 30.8–34.1) and differed between the group vaccinated at < 2 years (29.1; 95%CI = 27.2–31.2) vs. at ≥2 years (40.3; 95%CI = 37.7–43.0; z = 8.4; *p* < 0.0001).

A booster dose was administered to 128/181 (70.7%) seronegative enrollees. Within this group, 118/128 (92.2%) individuals were re-tested for anti-rubella IgG and 115/118 (97.5%; 95%CI = 92.7–99.5%) were determined to have seroconverted; 3/115 (2.6%; 95%CI = 0.5–7.4%) remained seronegative. The seroconversion rate after a booster dose differed between the group vaccinated at < 2 years (*n* = 87/87; 100.0%; 97.5%CI = 95.8–100.0%) and at ≥2 years (*n* = 28/31; 90.3%; 95%CI = 74.2–98.0%; *X*^2^ = 8.6; *p* = 0.017). The anti-rubella IgG geometric mean titer after a booster dose was 40.4 (95%CI = 34.9–46.8), and did not significantly differ between the group vaccinated at < 2 years (38.2; 95%CI = 32.5–45.1) vs. at ≥2 years (47.2; 95%CI = 33.9–65.8; z = 1.5; *p* = 0.137). After the administration of an additional dose of MMR vaccine to the seronegative group, re-testing showed that 33.3% had seroconverted. Overall, among those who were seronegative at baseline, 100.0% (97.5%CI = 98.0–100.0%) seroconverted after one or two booster doses.

The results of the univariate and multivariate logistic regression analyses of the association between seropositivity at enrollment and the tested determinants are presented in Table 1. According to the univariate logistic regression, seroconversion after a booster dose was associated with age at enrollment (OR = 0.73; 95%CI = 0.56–0.96; z = 2.3; *p* = 0.023), age at the second dose of rubella/MMR vaccine (OR = 0.81; 95%CI = 0.68–0.96; z = 2.5; *p* = 0.013), time from the first rubella/MMR vaccine to the booster dose (OR = 1.5; 95%CI = 1.1–2.0; z = 2.5; *p* = 0.014), and time from the second rubella/MMR vaccine to the booster dose (OR = 1.45; 95%CI = 1.04–2.01; z = 2.2; *p* = 0.027). There were no associations between the outcome and any of the other determinants (*p* > 0.05). In the multivariate logistic regression model there was no association between the outcome and any of the determinants (p > 0.05).

**Table 1:** Univariate and multivariate analyses of the determinants of rubella IgG seropositivity at study enrollment

Determinant	Univariate logistic regression		Multivariate logistic regression	
	OR	95%CI	aOR	95%CI
Sex				
• female	1.0	–	1.0	–
• male	0.5	(0.4–0.7)	0.5	(0.4–0.7)
Age at enrollment (years)	0.95	(0.90–1.01)		
Age at the time of first MMR vaccine				
• < 2 years	1.0	–	1.0	–
• ≥2 years	1.7	(1.2–2.4)	1.5	(0.9–2.4)
Age at the time of second MMR vaccine (years)	1.05	(1.00–1.10)	0.96	(0.89–1.04)
Time from the first to the second vaccine during the basal cycle (years)	0.99	(0.96–1.04)		
Time from the first MMR vaccine to the measurement of the antibody titer (years)	0.93	(0.89–0.97)	0.99	(0.93–1.06)
Time from the second MMR vaccine to the measurement of the antibody titer (years)	0.90	(0.85–0.95)	0.89	(0.80–0.99)

Chi-squared = 811.4; *p* = 0.785

*OR* odds ratio, *CI* confidence interval

**Fig. 2:**
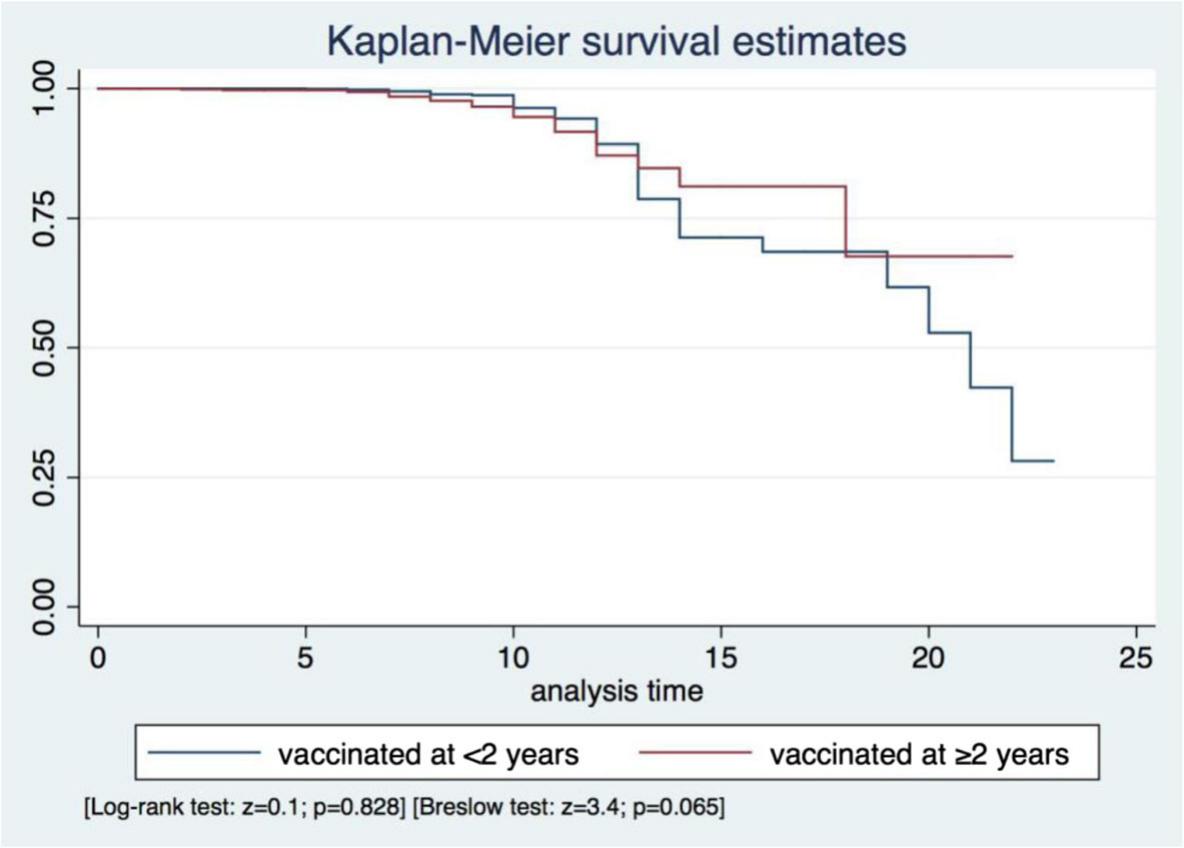
Kaplan-Meier-based estimates of rubella IgG protective antibody survival per age at first rubella/MMR vaccine (<2/≥2 years)

The average PAS was 10.2 ± 3.0 years (range = 0.0–23.0), the estimated time to the loss of anti-rubella IgG in 25% of the fully vaccinated 14 years (95%CI = 14–19), and the IRR per person-year of seronegativity 0.009 (95%CI = 0.008–0.010). There was no difference in the estimated PAS between groups (*p* > 0.05; Fig. 2). The IRR of seronegativity was 0.009 (95%CI = 0.008–0.011) in the group vaccinated at < 2 years and 0.007 (95%CI = 0.005–0.010) in the group vaccinated at ≥2 years, corresponding to an IRR of 0.8 (95%CI = 0.5–1.1; *p* = 0.048).

The univariate Cox semiparametric regression showed that PAS was associated with sex (hazard ratio [HR] = 1.8; 95%CI = 1.3–2.4; z = 3.8; *p* < 0.0001) and the age at the second routine vaccine (HR = 1.2; 95%CI = 1.1–1.3; z = 7.2; p < 0.0001). There was no association between the outcome and any of the other determinants (p > 0.05). The final multivariate Cox semiparametric regression model, stratified by age at the second dose of routine vaccine, showed an association with sex (HR = 1.7; 95%CI = 1.2–2.3; z = 3.4; *p* = 0.001; Table 2).

**Table 2:** Univariate and multivariate Cox semiparametric regression analysis of risk predictors of rubella IgG protective antibody survival

Determinant	Univariate Cox regression		Multivariate Cox regression model^a^	
	HR	95%CI	aHR	95%CI
Sex				
• female	1.0	–	1.0	–
• male	1.8	(1.3–2.4)	1.7	(1.2–2.3)
Age at enrollment (years)	0.96	(0.90–1.02)		
Age at the time of first MMR vaccine				
• <2 years	1.0	–		
• ≥2 years	1.04	(0.74–1.47)		
Age at the time of second MMR vaccine (years)	1.2	(1.1–1.3)		

*HR* hazard ratio, *aHR* adjusted hazard ratio

^a^Stratified by age at the time of second dose of MMR vaccine

Regarding the safety of the vaccine, in the 1-month follow-up there were no serious and/or long-term adverse reactions. The most commonly reported reactions were pain at the injection site and mild fever whereas laterocervical lymphadenopathy was a rare occurrence. All adverse events resolved without sequelae.

## Discussion

In our study of 2000 immunized medical students and residents, anti-rubella IgG could not be detected in 9% despite their having previously received two doses of rubella/MMR vaccine. In this group, one or more booster doses were needed to achieve seroconversion.

The percentage of seroconversion after one booster dose was high (97%) and after two doses was 100%. Female sex was associated with a longer persistence of anti-rubella IgG. Sex-based differences in the responses to vaccines have been studied. The various reports cited immunological, hormonal, genetic, microbiotic, and environmental differences between males and females as also potentially affecting the outcome of vaccination and found that males were generally less immunoresponsive than females [20–22].

In our study population, the time between vaccine administration and measurement of the antibody titer was a determinant of the detection of persisting circulating antibodies. Specifically, we found that antibody levels tended to decline ~ 10 years after completion of the basal cycle whereas in another study the time to a decline was 15 years [12].

To our knowledge, only a 2018 study investigated the management of non-responders to rubella vaccine. The introduction of a third booster dose in fully vaccinated but not seroprotected individuals has been investigated only by McLean et al. [23]. Those authors showed that the administration of a third dose of MMR vaccine to young adults without circulating anti-rubella IgG resulted in a seroconversion rate of 94%, similar to the results of our study of two booster doses. International public health institutions do not currently recommend pre-vaccination screening for HCWs nor a third MMR dose in susceptible health personnel. In 2011, the Advisory Committee on Immunization Practices (ACIP) [24] concluded that for HCWs who do not have adequate presumptive evidence of rubella immunity, pre-vaccination antibody screening is not necessary unless the medical facility considers it cost effective. Furthermore, an additional dose of MMR vaccine for the prevention of rubella is not recommended in a HCW with at least one documented dose of rubella vaccine but who is serologically negative or has equivocal rubella titer results. Our study showed that even in the fully vaccinated ~ 10% will not have detectable levels of circulating antibodies. The immunological status of these individuals is unclear [25, 26], as nosocomial infections in vaccinated HCWs have been reported [27]. Based on those findings, our management strategy should be carefully evaluated and the efficacy of additional doses of MMR in the prevention of rubella in the immunized population without circulating IgG investigated in further studies.

The management of non-responder HCWs with respect to the MMR vaccine must be considered in future decisions on vaccination strategies. Among the strengths of our study are the large sample size and the inclusion of a comparison of the age at first routine vaccine. Unfortunately, however, we could not analyze the immune status of the studied HCWs in relation to the type of vaccine (Monocomponent/MR/MMR/MMRV) nor could we determine whether they had ever been exposed to rubella. A repeat evaluation of the long-term immunogenicity of the rubella vaccine and the management of non-responders should be considered in studies with a large sample size and an extended follow-up time. In addition, the long-term trend in immunogenicity and the cost-efficiency of an effective management strategy should be evaluated.

## Conclusions

The CDC recommends that healthcare providers routinely assess women of childbearing age for evidence of rubella immunity and vaccinate those lacking acceptable evidence of immunity [28]. In occupational medicine, the serological evaluation of rubella must become routine, especially in HCWs whose jobs include contact with pregnant women, in order to reduce the risk of rubella virus circulation in this population. In Italy, the vaccination of HCWs is a highly debated topic. In 2018, the Emilia-Romagna Regional Authority approved a regional law that makes measles, mumps, rubella and varicella vaccines mandatory for susceptible HCWs working in operative units where the risk of infection is high (oncology, neonatology, gynecology, infectious disease, etc.), in order to prevent nosocomial infections and to protect patients’ health [29]. In the same year, with the goal of increasing vaccination compliance among HCWs, the Apulian Regional Authority approved a regional law making vaccinations mandatory for healthcare personnel; however, the Italian Government contested the law to the Constitutional Court [30]. More recently, the Italian Ministry of Health proposed mandatory vaccination of everyone, including HCWs, entering public competitions, but this proposal awaits adoption. Implementing such measures in health facilities could contribute to a reduction of both rubella cases in adults and congenital rubella.

## Data Availability

The datasets generated and/or analyzed for this study are not publicly available due to privacy issues but are available from the corresponding author on reasonable request.

## Abbreviations

CDC: Center for Disease Control and Prevention
GIAVA: Regional Immunization Database
HCWs: Healthcare workers
MMR: Measles, mumps, rubella
WHO: World Health Organization
